# Spatial distribution and ecological risk assessment of heavy metals in karst soils from the Yinjiang County, Southwest China

**DOI:** 10.7717/peerj.12716

**Published:** 2022-02-01

**Authors:** Ruiyin Han, Zhifang Xu

**Affiliations:** 1 Institute of Geology and Geophysics, Chinese Academy of Sciences (CAS), Beijing, China; 2 University of Chinese Academy of Sciences, Beijing, China; 3 Chinese Academy of Sciences, Center for Excellence in Life and Paleoenvironment, Beijing, China

**Keywords:** Contamination assessment, Distribution characteristics, Land-use types, Karst area, Soil heavy metals

## Abstract

**Background:**

Soil heavy metals (HMs) under different land-use types have diverse effects, which may trigger the ecological risk. To explore the potential sources of HMs in karst soils, the spatial distribution and geochemical behavior of HMs based on different land-use types are employed in this study.

**Methods:**

Soil samples (*n* = 47) were collected in three suites of karst soil profiles from the secondary forest, abandoned cropland and shrubland in Yinjiang, Southwest China. The concentrations of Ni, Mn, Cr, Pb, Cd and Mo were determined to give a comprehensive understanding of the possible sources of these HMs and evaluate the potential ecological risk in Yinjiang County.

**Results:**

The mean concentrations of HMs in all profiles followed the same order: Mn > Cr > Ni > Pb > Mo > Cd. Meanwhile, the concentrations of most HMs roughly increased with the depth. Additionally, the concentrations of HMs were mostly correlated with soil pH and SOC, rather than with clay and silt proportions. By contrast, with the enrichment factors (EF), geo-accumulation (I_geo_) and potential ecological risk index (PERI) of HMs in soil under different land-use types, the results indicated that these HMs exhibited non-pollution (I_geo_ < 0) and no ecological risk (PERI < 30) to human health in soils of Yinjiang County.

**Conclusions:**

The distribution of HMs is dominated by weathering in the karst area, and the effects of agricultural inputs on the enrichment of soil HMs in Yinjiang County are limited. This further state that the arrangement of the local agricultural structure is reasonable.

## Introduction

Soil acts an important sink of heavy metals (HMs) in the Earth’s surface system. There are two ways for soil to accumulate HMs: (i) natural inputs from the weathering of continental rocks, and (ii) anthropogenic sources such as industrial production, atmospheric precipitation and agricultural activities (Taylor et al., 2010; Wei & Yang, 2010). As a vital environmental media, soil can be directly affected by human activities, including farming activities, mining development and smelting (Qiu et al., 2016; Skierszkan et al., 2016; Wang & Zhang, 2007). Soil environment, in turn, affects human health in multifarious ways. The poisonous Mo, Cd and Pb metals in soil can be easily absorbed by crops, resulting in a high chronic carcinogenic risk for human beings (Demir, 2021; Xiao et al., 2017). The intake of proper quantity of HMs is essential for the living organisms growing, whereas the excessive intake of HMs will provoke detrimental effects on vegetation, animals and human bodies (Sawut et al., 2018; Taylor et al., 2010). The soil adsorption of Cr is limited because Cr is mostly available in water-soluble or exchangeable from in soil (Liu et al., 1990). As a migratory pollutant, Cr could very easily affect the resident water environment by polluting groundwater. The compounds of Mn and Ni may also be absorbed by plants and forage crops, ultimately into the body of herbivores and humans (Bashir, Ahmad & Khan, 2020; Fardous et al., 2011). Excessive HMs and their interaction can also aggravate bioavailability and ecological risk (Lago-Vila et al., 2017). Accordingly, the accumulation degree of HMs (*e.g.*, Mn, Ni, Cr, Pb, Cd and Mo) in soils can indicate soil environment pollution level and ecological risk.

The information containing in a suit of soil profile can reflect the soil physical and chemical processes at a specific site (Liu et al., 2016b; Vodyanitskii & Yakovlev, 2011). Previous researches have mainly focused on the spatiotemporal variations and the contamination of HMs in human-affected regions, for examples, sewage irrigation area, polluted farmland and mining area (Khanal et al., 2014; Kong et al., 2018; Liu et al., 2016a; You et al., 2015). However, the studies of HMs characteristics in soil profile were mostly aimed at a single land-use type (Balabane et al., 1999; Vodyanitskii & Yakovlev, 2011). Little is known about the vertical distribution of HMs based on different land-use types under the same geological background, especially for the soils in the karst area. The manner and degree of human disturbance are closely associated with land-use types, which can affect the spatial distribution of HMs in soil profiles. The research on HMs characteristics in soil profiles under different land-use types is almost negligible and thus poorly documented. Accordingly, it is of vital importance to investigate the vertical characteristics of HMs under different land-use patterns and human interferential degrees in the karst regions.

The karst ecosystem belongs to a highly fragile ecosystem that can be easily affected by anthropogenic activities (Han et al., 2020). The slight discrepancy in different soil types may be more prominent in the karst region (Liu et al., 2013; Parise, De Waele & Gutierrez, 2009). In the karst region, the high-rate of rock weathering and low-rate of soil formation can result in a strong spatial of soil distribution and chemical composition in soils (Gao et al., 2013). Even though at the similar depth, the physicochemical properties and HMs concentrations in the soils derived from different locations likely show substantial discrepancies (Gregorauskiene & Kadunas, 2006). Moreover, the soil was mainly developed from limestone in the karst area of southwest China, and sizable amounts of HMs were released due to its unique geochemical process (Wen et al., 2020; Zhao et al., 2015). Naturally, the concentrations of Mn, Ni, Cr, Pb, Cd and Mo in karst soils are higher than other area which are not developed from limestone (Wen et al., 2020). The migration ability of HMs is stronger with high porosity and heterogeneous distribution in karst soil (Tao et al., 2020). The high concentrations of HMs are harmful to the large soil environment area through surface water and groundwater flow due to the unique hydraulic and hydrogeological characteristics of karst area (Huang et al., 2020; Reimann & Caritat, 2000). Therefore, it is necessary to analyze the behavior of HMs in the karst area showing potentially higher background concentration.

In Yinjiang County, in addition to weathering and pedogenic processes, agricultural activities play a significant role in regulating the geochemical behaviors of HMs in soils (Huang et al., 2017; Xu et al., 2017b). Therefore, the purposes of this study were to: (1) explore the vertical distribution of HMs in the profiles under different land-use types; (2) determine the influence of rock weathering processes and anthropogenic inputs on the distribution of HMs in the soils under different land-use types; and (3) evaluate the ecological risks of HMs in karst areas by the enrichment factor (EF), geo-accumulation index (I_geo_) and potential ecological risk index (PERI). This study is desirable to extend the knowledge of the migration process of HMs in soil under different land-use types soils in karst area and evaluate the possible influence of interferential degrees from human. The HMs results in this study can supply the data supporting soil management for soil quality and sustainability.

## Materials & Methods

### Study area

**Figure 1:**
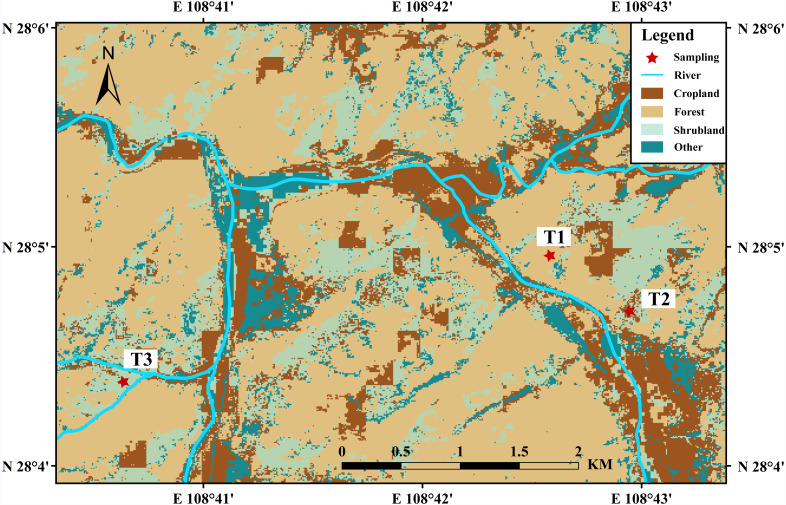
Land-use types profiles sites and in the study area.

The study area is located in the Yinjiang County (Fig. 1), a karst region in Guizhou Province, of Southwest China. The study area lies between 27°35′–28°21′N and 108°18′–108°48′E, with above 454,000 permanent resident population. The Yinjiang County is dominated by the subtropical monsoon climate, with the variation in temperature from −9 °C to 39.9 °C (Xu et al., 2017b). Rainfalls are mainly concentrated from April to September, with the annual precipitation of 1,057–1,268 mm (Xu et al., 2017a). The rock exposed in the Yinjiang County is dominated by the Permian and Triassic carbonates, with a rocky desertification area of 11783.06 hm^2^ (Huang et al., 2017; Li, 2018). The elevation decreases from southeast to northwest, a typical karst trough valley with a relative elevation exceeding 2,000 m (Li, 2018). The study area is far away from urban cities and diggings and mostly covered by cropland (Huang et al., 2017). The agricultural areas in Yinjiang County accounted for nearly 30% of the total area with the main crops being corn and sweet potatoes (TBS, 2017), and the forest area accounted above 60% of the total area with the dominant vegetation of *Platycarya strobilacea Sieb.et Zucc., Melia azedarach L.* and *Quercus fabri Hance*. The vegetation of shrubland is mainly cultivated with *Pyracantha fortuneana, Castanea mollissima, Lindera communis*. The main soil types of Yinjiang county are Mollic Inceptisols Soil Survey Staff, 2010, which are calcareous soils derived from limestone rocks.

### Sample collection

A total of 47 soil samples were collected during September 2016 in the Yinjiang County, from the three soil profiles in secondary forest land (T1, *n* = 20), abandoned cropland (T2, *n* = 16) and shrubland (T3, *n* = 11), respectively. Due to the strong spatial heterogeneity in soil properties, particularly at the vertical direction, three duplicate soil profiles of less than 1 meter were selected at each sampling sites. Moreover, the results were presented as an average of three samples derived from the three duplicate profiles at the same depth in the present study. The detailed descriptions of soil profiles are shown in Table 1.

**Table 1:** Geographic information, dominant vegetation and profile characteristic at the soil sites.

Profile	Location	Altitude (m)	Thickness (cm)	Land-use types and primary vegetation	Profile descriptions
Secondary forest (T1)	28°04′57.64″N 108°42′31.01″E	838	160	Subtropical evergreen broad–leaved secondary forest, interspersed with *Platycarya strobilacea Sieb.et Zucc., Melia azedarach L., Quercus fabri Hance. etc.*	0–5 cm: Gray soil, abundant plant roots, and debris. 5–85 cm: Yellow clay and silt, few small stone particles. 85–160 cm: Relatively uniform brawn to red soil, connect to bedrock.
Abandoned cropland (T2)	28°04′48.35″N 108°42′58.22″E	892	130	Sloping farmland, cultivation history is about 50 years, the main crops are corn and potatoes, which have been abandoned 3 years before sampling	0–25 cm: Yellow soil, few plant residues, and small stone particles. 25–110 cm: Relatively uniform yellow fine silt. 110–130 cm: Yellow brawn to red brawn soil, more stones.
Shrubland (T3)	28°04′22.68″N 108°40′37.62″E	776	70	Native shrub grass slope, the main plants are *Pyracantha fortuneana, Castanea mollissima, Lindera communis*, and interspersed with less *Cunninghamia lanceolata, Pinus massoniana Lamb*, *etc.*	0–10 cm: Black soil, abundant humus, few stones. 10–25 cm: Gray soil, abundant stones. 25–70 cm: Yellow soil, few stones, connect to bedrock.

### Soil analyses

Soil samples were air-dried and sieved through a two mm sieve after removing big litters and stones. For subsequent analysis, soils were entirely grounded to around 200 mesh. Soil particles were categorized into three groups including clay (<2 µm), silt (2 µm to 50 µm) and sand (50 µm to 2,000 µm) according to USDA Soil Taxonomy (Soil Survey Staff, 2010). Soil pH was measured using glass electrode in the 1:2.5 soil–water suspension with a precision of ±0.05. Soil powders were digested with HNO_3_–HF–HClO_4_ (Li et al., 2022; Li et al., 2020; Liu, Han & Li, 2021). The concentrations of Al, Cr, Mn were determined by ICP–OES (Optima 5300DV; Perkin Elmer, Waltham, MA, US) and the concentrations of Ni, Mo, Cd, Pb were analyzed by ICP–MS (Elan DRC–e; Perkin Elmer, Waltham, MA, US) in the Institute of Geographic Sciences and Natural Resources Research, CAS at precision ±5%. Quality control and quality assurance were performed by the procedural blank and standard reference material (GBW07447 and GBW07449).

### Data calculation

#### Index of enrichment factor

As the indicator in various environmental media, the enrichment factor (EF) and the Geo–accumulation Index (I_geo_) widely employs to quantify the accumulation and contamination of metallic elements through calculating the soil exchangeable fractions (Barbieri, 2016; Zeng, Han & Yang, 2020).

The indexes of EF are usually calculated by the normalized concentration of a metal relative to its reference concentration (Barbieri, 2016; Mazurek et al., 2017). The representative element used in several studies is Al due to its insusceptible property (Ackermann, 1980; Blaser et al., 2000). The formula of EF is shown as:

(1) \begin{eqnarray*}\mathrm{EF}= \frac{{ \left( \mathrm{M}/\mathrm{Al} \right) }_{\mathrm{S}}}{{ \left( \mathrm{M}/\mathrm{Al} \right) }_{\mathrm{B}}} \end{eqnarray*}

where M means the concentrations of metal (mg kg^−1^), and S means soil samples. And calculated the (M/Al)_B_ ratio based on the HMs and Al values in the average soils of Guizhou Province (China Environmental Monitoring Station, CEMS)(1990). Barbieri (2016) categorized the EF values into five grades (Table 2).

**Table 2:** The classification of EF values and I_geo_ values.

EF	Soil quality	I_geo_	Soil quality
EF < 2	Negligible enrichment	I_geo_ < 0	Non-pollution
2 ≤ EF < 5	Moderate enrichment	0 ≤ I_geo_ < 1	Minor pollution
5 ≤ EF < 20	Significant enrichment	1 ≤ I_geo_ < 2	Moderate pollution
20 ≤ EF < 40	Severe enrichment	2 ≤ I_geo_ < 3	Moderate to severe pollution
EF ≥ 40	Extremely severe enrichment	3 ≤ I_geo_ < 4	Severe pollution
		4 ≤ I_geo_ <5	Severe to extreme pollution
		I_geo_≥ 5	Extreme pollution

#### Index of Geo–accumulation

The Geo–accumulation Index (I_geo_) is extensively employed to evaluate anthropogenic contamination levels (Nazeer, Hashmi & Malik, 2014; Zoller, Gladney & Duce, 1974). Müller (1971) defined the formula of I_geo_ as:

(2) \begin{eqnarray*}{\mathrm{I}}_{\mathrm{geo}}=\log \nolimits 2 \left( {\mathrm{S}}_{\mathrm{M}}/1.5{\mathrm{R}}_{\mathrm{M}} \right) \end{eqnarray*}

where S_M_ represents the concentrations of HMs in samples; R_M_ represents the reference value for HMs in Guizhou Province (China Environmental Monitoring Station, CEMS)(1990), and the constant 1.5 is applied to eliminate the lithological fluctuations (Barbieri, 2016). Accordingly, the values of I_geo_ are separated into seven classes (Table 2) from non-pollution to extreme pollution (Müller, 1971).

#### Index of potential ecological risk

Hakanson (1980) originally proposed the potential ecological risk index (PERI) to effectively appraise the ecological risk of HMs in sediment or soil. Extensive studies have applied PERI to estimate the potential ecological risk and pollution level triggered by single or multiple HMs (Aboubakar et al., 2021; Gujre, Rangan & Mitra, 2021; Sun et al., 2010). The (3)–(5) to calculate PERI are as:

(3) \begin{eqnarray*}{\mathrm{C}}_{\mathrm{c}}^{\mathrm{i}}={\mathrm{C}}_{\mathrm{ s}}^{\mathrm{i}}/{\mathrm{C}}_{\mathrm{ r}}^{\mathrm{i}}\end{eqnarray*}

(4) \begin{eqnarray*}{\mathrm{E}}_{\mathrm{f}}^{\mathrm{i}}={\mathrm{T}}_{\mathrm{ f}}^{\mathrm{i}}\times {\mathrm{C}}_{\mathrm{ c}}^{\mathrm{i}}\end{eqnarray*}

(5) \begin{eqnarray*}\mathrm{RI}=\sum _{\mathrm{i}=1}^{\mathrm{n}}{\mathrm{E}}_{\mathrm{ f}}^{\mathrm{i}}\end{eqnarray*}

where C_c_^i^ indicates the contaminated factor of each heavy metal, C_s_^i^ represents the measured concentration of HMs in soils, C_r_^i^ represents the reference value for HMs in the average soils of Guizhou Province (China Environmental Monitoring Station, CEMS)(1990). E_f_^i^ indicates the potential ecological risk of each heavy metal, T _f_^i^ represents the toxic response factor of respective HMs, and RI indicates the comprehensive potential ecological risk of soil HMs (Hakanson, 1980). T_f_^i^ values of Mn, Cr, Pb, Ni and Cd were obtained from Xu et al. (2008) and were 1, 2, 5, 5, 30, respectively. Unfortunately, the toxicity response factor of Mo is indefinite.

Based on the contaminated degree of single heavy metal, the values of C _c_^i^ and E _f_^i^ are classified into five classes and the values of RI are divided into 4 classes by the comprehensive value of the PERI of multiple HMs (Gujre, Rangan & Mitra, 2021; Qiu et al., 2016). The specific evaluation indicators and classes are shown in Table 3.

**Table 3:** The corresponding relationships of C_c_^i^, E_f_^i^, RI, and contaminated degree.

C_c_^i^	Contaminated degree	E_f_^i^	Contaminated degree	RI	Contaminated degree
C_c_^i^≤ 0.7	Great	E_f_^i^ < 40	Slight ecological risk	RI < 150	Slight ecological risk
0.7 < C_c_^i^≤ 1.0	Safety	40 ≤ E_f_^i^ < 80	Moderate ecological risk	150 ≤ RI < 300	Moderate ecological risk
1.0 < C_c_^i^≤ 2.0	Slight contamination	80 ≤ E_f_^i^ < 160	High ecological risk	300 ≤ RI <600	High ecological risk
2.0 < C_c_^i^≤ 3.0	Moderated contamination	160 ≤ E_f_^i^ < 320	Heavy ecological risk	RI ≥ 600	Heavy ecological risk
C_c_^i^> 3.0	Heavy contamination	E_f_^i^≥ 320	Extremely ecological risk		

The relationship between different HMs and soil properties was identified by linear- regression analysis, with the determination of the coefficient R and *p*-values by SPSS 25.0 (IBM SPSS Statistics, Chicago, IL, US). The graphics were completed by Origin 2017 (OriginLab, Northampton, MA, USA).

## Results

### Soil properties

Soil properties (*e.g.*, soil pH and soil particle distribution) are the influencing factors that regulate the concentrations of HMs in natural soils (Wang & Zhang, 2007; Zhang et al., 2018). The variations of soil properties in all profiles are summarized in Table 4. The values of soil pH T1 profile: 7.1–7.9, T2 profile: 4.8–5.2 and T3 profile: 6.3–7.0) in the three profiles have been reported by Han & Xu (2021). Soil silt particle accounted for the largest portion (mean: 85.87% in T1profile; 75.12% in T2 profile; 85.25% in T3 profile), and the second largest was clay (mean: 12.96% in T1profile; 15.50% in T2 profile; 12.09% in T3 profile) in all profiles (Han & Xu, 2021). The contents of soil SOC in three profiles ranged from 0.38% to 4.2% in T1 profile, 0.51% to 1.7% in T2 profile and 0.76% to 10.8% in T3 profile.

**Table 4:** Soil properties and HMs concentrations (mg/kg) in soil samples of the three profiles. The data of soil pH and soil particle proportion were reported by Han & Xu, (2021).

Profiles	Depth (cm)	pH	SOC (%)	Clay (%)	Silt (%)	Sand (%)	Cr	Mn	Mo	Ni	Pb	Cd
Secondary forest (T1)	0	7.2	4.22	13.58	83.97	2.45	79.75	683.8	2.92	41.47	31.73	0.83
	5	7.7	1.73	14.34	84.31	1.35	81.66	642.6	3.12	40.38	28.81	0.70
	10	7.7	1.63	13.38	84.74	1.88	84.42	694.6	3.52	40.47	30.24	0.69
	15	7.7	1.27	14.68	84.34	0.98	76.58	668.9	2.78	37.73	27.59	0.46
	20	7.7	0.85	12.92	85.79	1.29	80.77	626.0	2.17	39.51	28.64	0.35
	30	7.7	0.75	14.41	84.46	1.13	74.99	606.8	2.17	38.23	28.16	0.34
	40	7.9	0.76	15.41	83.92	0.67	73.59	583.5	1.97	40.42	28.69	0.33
	50	7.9	0.94	13.99	85.07	0.94	61.94	557.6	1.67	34.36	24.19	0.32
	60	7.8	0.71	14.09	84.93	0.99	62.86	647.6	1.86	41.48	29.06	0.33
	70	7.5	0.71	15.99	83.30	0.72	50.45	447.6	1.95	41.57	29.70	0.34
	80	7.7	0.48	14.55	84.82	0.63	55.69	397.5	1.77	40.63	28.23	0.28
	90	7.5	0.41	12.38	86.70	0.92	54.19	342.9	2.24	47.32	28.43	0.24
	100	7.3	0.46	12.60	86.54	0.86	59.71	395.2	2.02	46.24	28.56	0.27
	110	7.3	0.39	12.66	85.79	1.55	56.82	417.2	1.60	41.61	27.37	0.29
	120	7.2	0.38	11.83	87.65	0.52	58.06	454.1	1.67	44.05	29.20	0.31
	130	7.1	0.40	11.00	87.90	1.09	57.14	433.5	1.71	48.48	29.44	0.31
	140	7.1	0.60	10.56	88.42	1.02	67.33	475.9	2.23	55.50	33.62	0.37
	150	7.2	0.47	9.07	88.96	1.97	63.65	507.8	2.28	54.06	34.85	0.34
	160	7.3	0.52	8.71	89.88	1.41	59.29	368.9	2.04	49.50	32.28	0.32
Bedrock							26.57	26.57	85.20	1.14	12.16	8.10
Abandoned cropland (T2)	0	4.8	1.77	10.12	75.46	14.43	59.05	298.8	1.37	31.21	29.60	0.45
	5	4.8	0.70	15.16	72.77	12.08	54.28	258.2	1.05	30.93	25.53	0.31
	10	4.9	0.70	14.05	75.12	10.83	53.73	283.9	1.04	28.91	25.51	0.30
	15	4.8	0.59	12.94	68.65	18.42	46.58	360.5	0.93	29.21	25.79	0.29
	20	5.0	0.58	14.10	75.28	10.63	51.29	331.9	0.91	28.39	25.05	0.26
	30	5.0	0.57	14.30	75.84	9.87	50.18	320.1	0.83	26.98	24.06	0.22
	40	5.1	0.57	16.12	75.86	8.02	49.89	328.3	0.83	27.69	24.00	0.23
	50	5.0	0.55	16.26	75.49	8.25	49.81	323.5	0.73	27.59	22.32	0.21
	60	5.1	0.57	17.90	74.49	7.61	44.70	311.7	0.74	26.07	22.76	0.22
	70	4.8	0.68	16.23	78.19	5.58	49.36	343.0	0.82	27.43	23.08	0.21
	80	4.8	0.73	17.04	77.30	5.66	53.41	314.4	0.81	26.88	23.59	0.19
	90	4.9	0.66	17.40	78.42	4.18	57.03	331.8	0.82	29.54	23.79	0.18
	100	4.9	0.83	16.37	79.63	4.00	49.92	315.8	0.84	26.71	22.94	0.21
	110	5.2	0.51	14.74	73.31	11.95	56.80	370.9	0.79	39.10	34.42	0.23
	120	5.1	0.53	17.82	73.61	8.57	61.58	778.4	0.79	42.00	31.85	0.19
	130	5.1	0.53	17.47	72.50	10.03	54.55	527.2	0.77	42.67	30.06	0.19
Shrubland (T3)	0		10.82				44.23	224.2	2.44	22.86	22.68	0.54
	5	6.3	7.07	10.45	85.99	3.56	41.27	208.6	2.48	24.51	20.71	0.44
	10	6.3	4.82	10.79	85.93	3.28	42.59	234.3	2.65	26.01	21.34	0.43
	15	6.4	3.91	12.16	85.10	2.75	39.97	164.6	2.05	20.63	14.80	0.25
	20	6.5	2.72	12.13	85.56	2.31	45.64	194.7	2.43	24.39	3.92	0.33
	30	6.6	1.90	13.33	84.35	2.32	47.41	215.7	3.02	29.76	17.58	0.34
	40	6.8	1.06	13.30	84.24	2.46	53.08	247.5	3.49	35.38	12.55	0.28
	50	7.0	0.84	12.87	85.26	1.87	52.80	236.7	3.84	37.69	19.47	0.26
	60	7.0	0.76	11.36	86.96	1.68	51.78	258.8	3.24	40.40	20.11	0.24
	70	7.0	0.90	12.39	83.90	3.71	57.70	283.8	2.55	37.07	14.14	0.25
Bedrock							26.49	62.69	0.60	9.20	4.17	0.06

### HMs in the soil profiles

**Figure 2:**
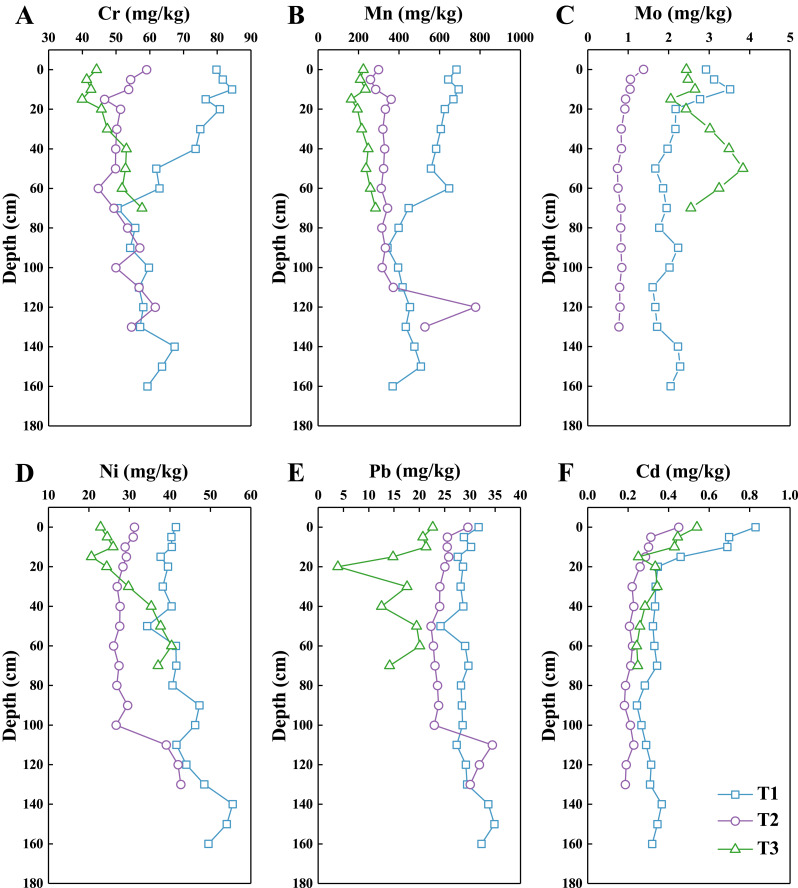
Vertical variation of HMs in the three soil profiles, including Cr (A), Mn (B), Mo (C), Ni (D), Pb (E), and Cd (F).

The vertical distributions of the six HMs (Mn, Ni, Cr, Pb, Cd and Mo) in the three soil profiles under different land-use types are presented in Fig. 2, and the concentration data are shown in Table 4. The concentrations of all HMs in Yinjiang County were higher than those in the upper continental crust but lower than the values from the Draft soil screening guidance reported by the EPA (OSWER 1993; Rudnick & Gao, 2003). Most of the concentrations of HMs are lower than the average soils of Guizhou Province (China Environmental Monitoring Station, CEMS)(1990). The high geological background values of HMs may be related to the regional geochemistry and the endogenous influence of the pedogenesis process in the Guizhou Karst area (Chen et al., 2019). A large amount of HMs is released into soils during the weathering of carbonate rocks, causing HMs “concentrated” in soil (Wu et al., 2020). The concentrations of metals in all profiles decreased in the following order: Mn (400.43 mg/kg) > Cr (57.28 mg/kg) > Ni (35.85 mg/kg) > Pb (25.34 mg/kg) > Mo (1.87 mg/kg) > Cd (0.33 mg/kg). The concentrations of most HMs in the T1 profile under secondary forest were the highest in the three profiles. The concentrations of most HMs tended to be similar in the bottommost soil

### Indexes of ecological risks assessment

Based on the calculation, the EF values of most HMs in soils from three profiles were less than 2. However, the EF values of Ni ranged from 2.16 to 3.90 in the T1 profile and from 1.89 to 2.86 in the T3 profile. The EF values of Pb were greater than 2 in more than one third of the T1 profile, while the EF > 2 was found in the bedrock. Except the sample at the 40 cm depth in the T3 profile, the I_geo_ values of all HMs in soils were lower than 0. Excluding for the C_c_^i^ values of Mo, Ni and Cd in the T1 profile and Mo in the T3 profile were greater than 1, the C_c_^i^ values of other HMs in soils were less than 1. Moreover, most soils showed that values of C_c_^i^ were less than 0.7, especially in the T2 profile. The RI values reveal that Cr, Mn, Ni, Pb and Cd were of slight ecological risk (RI < 60) in the three profiles.

## Discussion

### Effects of soil particles on HMs

Generally, HMs concentrations are significantly correlated with soil particle distribution (Probst et al., 2003). The higher concentrations of HMs in soil are always related to a larger proportion of clay because of the larger specific surface, which tend to increase the absorption capacity of HMs (Jaradat et al., 2009). Although the clay contents in the T2 profile was relatively high, the study area soils were silt loamy texture and the clay contents were lower than 20% in three profiles. The adsorption capacity for the HMs is relatively weak.

The phenomenon of the rapid vertical migration of water during irrigation and rainfall was always found in cropland due to higher heterogeneity in cropland soil properties such as preferential flows (Brusseau & Rao, 1990). Clay is an important carrier of HMs. Thus, the preferential flow also promotes the translocation of adsorbed HMs by affecting the migration of fine particles (Zhang, 2005). In the process of transporting the solution by the preferential flow in soil profile, the chemical composition is stable (Zhang et al., 2016a; Zhang et al., 2016b). In recent years, several studies have found that heavy metals migrate rapidly from the soil surface to the deep soils through the soil preferential flow (Knechtenhofer et al., 2003; Zhang et al., 2016a; Zhang et al., 2016b). The preferential flow might affect the vertical migration of HMs in the T2 profile. In contrast, some studies suggest that the contribution of preferential flow in HMs migration is limited (Allaire et al., 2002; Zhang et al., 2016b). We also observed weak correlation between the size distributions of soil particles and HMs concentrations in this study. It can be inferred that the effect of soil particles is limited on the distribution of HMs in the study soils.

### Effects of soil organic carbon on HMs

**Figure 3:**
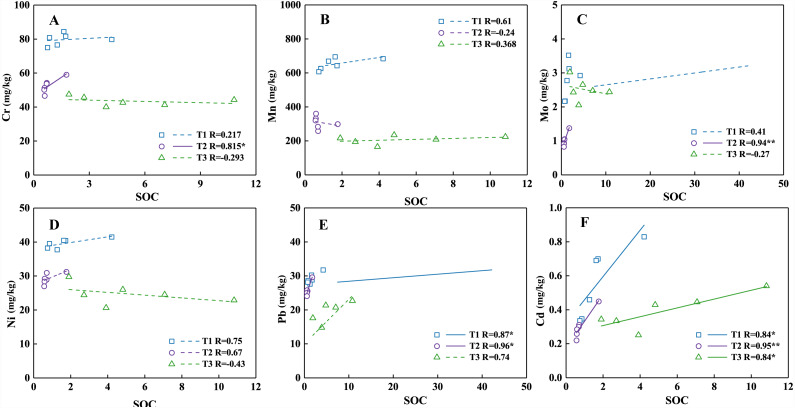
Correlation analysis of Cr (A), Mn (B), Mo (C), Ni (D), Pb (E), and Cd (F) with soil SOC in 0–30 cm of the three profiles. Asterisks: ** represents *p* < 0. 01; * represents *p* < 0.05.

SOC is one of the most important properties affecting HMs as the humus could easily coordinate or chelate with HMs by some functional groups (Dijkstra, 1998). The correlation analysis between SOC and HMs in 0–30 cm soil layers are presented in Fig. 3. Many studies indicated that the concentrations of HMs show a positive correlation with SOC in the various types of soils including in karst area (Balabane et al., 1999; Mazurek et al., 2017; Zhang et al., 2019). HMs can easily form stable compounds with the soil organic matter (SOM) (Mazurek et al., 2017). For example, there was strong correlations between SOC and Pb, Cd in the T1 profile (Figs. 3E, 3F), Cr, Mo, Pb, Cd in the T2 profile (Figs. 3A, 3C, 3E, 3F), and Cd in T3 profile (Fig. 3F). The contents of SOC in shrubland are possibly enriched in the surface soil, and decrease obviously with the depth in the surface soil due to grazing (Hiernaux et al., 1999). This phenomenon was also found in T3 profile, and the highest content of SOC was found in T3 profile. However, the distribution of SOC contents in the T2 profile is almost constant. And the contents of HMs almost have no fluctuations which are similar with the distribution of SOC in T2 profile, and present the great correlation between the contents of HMs and SOC. Generally, the content of SOC recovering difficultly in the abandoned cropland for the short term (Liu, Han & Li, 2021). The concentrations of HMs almost fluctuated moderately in the T2 profile (abandoned cropland), which may have resulted from the distribution of SOC.

The HMs can be strongly complexed with the organic matter because of the negative charges on its surface (Marks et al., 2015). The chelates formed by HMs and organic compounds may increase the availability of metals to plants or reduce their bioavailability to regulate the activities of HMs in soil (Dijkstra, 1998; Zhang et al., 2018). The absorption capacity of SOC to Cd, Mo and Pb is relatively large, thus may reduce the migration and increase the accumulation in soil (Dumat et al., 2006). However, most of the complexes formed by organic matter and Ni are humic acid, which will reduce the content of Ni in soil (Chimitdorzhieva, Nimbueva & Bodeeva, 2012). Therefore, the effects of SOC on HMs distribution in soils under different land-use types are mixed. There are multiple factors acting on the distribution of HMs. While the SOC has an important effect, it may not be the dominant factor.

### Effects of soil pH on HMs

**Figure 4:**
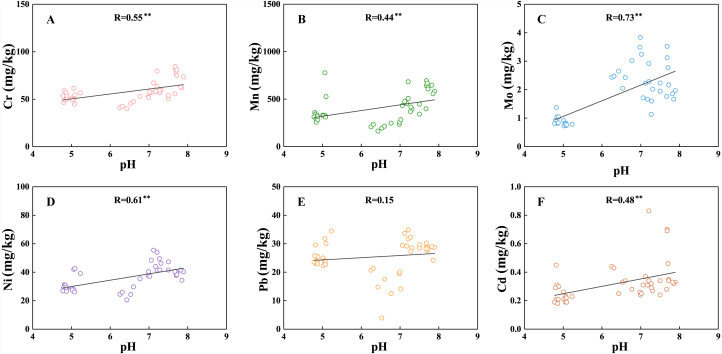
Correlation of soil pH with Cr (A); (B) Mn; (C) Mo; (D) Ni; (E) Pb; (F) Cd in all soils. Asterisks: ** represents *p* < 0. 01; * represents *p* < 0.05.

The concentrations of the six HMs were positively correlated with soil pH, and their correlation analysis is presented in Fig. 4. In the natural environment, the geochemical behaviors of trace elements are dominantly affected by pH (Yang et al., 2018). The changes of soil pH will directly or indirectly affect the soil adsorption of HMs by affecting the stability of complexes, oxide and organic material surface negative charge, hydrolysis of HM ions, the formation of ion pairs, *etc.* (Rieuwerts et al., 1998; Sauvé, McBride & Hendershot, 1997). The negative charge on the organic matter and clay minerals surface is likely to increase with a high pH, which further enhances the adsorption capacity and the complexes stability of HMs (Markiewicz-Patkowska, Hursthouse & Przybyla-Kij, 2005; Semerjian & Ayoub, 2003). In addition, HMs will enrich in soil under high pH environment because of decreasing metal availability (Sparks, 2003). Generally, the distribution of HMs is mainly controlled by adsorption reaction under acidic conditions, while the precipitation reaction of HMs and hydroxides or carbonate account for a dominant proportion in medium-alkaline conditions (Ottosen, Hansen & Jensen, 2009). The relationship between Cr, Mo, Mn, Ni and Cd and soil pH presented similar relation under three soil profiles. With the lower pH and SOC content, the adsorption capacity of soil to HMs is lower in the T2 profile. The soil pH values and concentrations of HMs in the T1 profile are the highest. The soil pH possibly plays an important role in regulating the concentrations of HMs in the T1 profile. It should be considered that the relationship of Pb and soil particle distribution, SOC, soil pH is weak. Result showed that the Pb were slightly enriched in the topsoil (0–5 cm) of three profiles, which may be related to the atmospheric deposition or fertilizer usage (Kong et al., 2018).

### Soil contamination assessment

**Figure 5:**
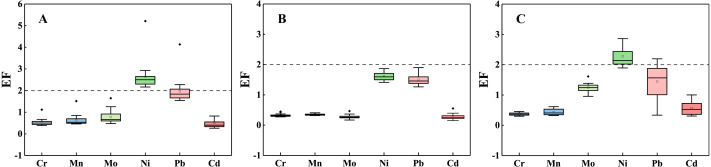
Variation of each EF_HMs_ value in the soils, including T1 profile (A), T2 profile (B), T3 profile (C).

The enrichment factors (EF) of HMs in the soils are quantified and displayed in Fig. 5. The mean EF values of most HMs in soils were less than 2, indicating that the enrichment of HMs in most soils was negligible. It is estimated that the characteristic of the geological material may regulate the HMs concentrations, and non-natural sources may contribute less. Only the EF values of all HMs in the soils of the T2 profile were less than 2. The agricultural activities may be limited to the accumulation of HMs in the T2 profile soil.

The higher EF values of Ni in the T1 and T3 profiles indicate that the element Ni was moderately enriched in the T1 and T3 profiles. The Ni concentrations in most soils are close to the background value of Guizhou Province. The difference among the three profiles may be related to the SOC. The study shows that the content of organic matter in abandoned farmland is significantly lower than that in normal vegetation-covered soil, furthermore, the content of SOC in soils will not return to the normal level in a short time after land abandoned (Liu, Han & Li, 2021). The EF higher values of Pb in the T1 profile suggest that the Pb of T1 profile are derived from weathering. The EF values of Pb greater than 2 was found in the shallow soil (0–5 cm) of the T3 profile, which may be caused by atmospheric deposition (Zhang et al., 2016a). Furthermore, the shallow soil is rich in organic matter which has better adsorption of Pb (Harter & Naidu, 1995).

**Figure 6:**
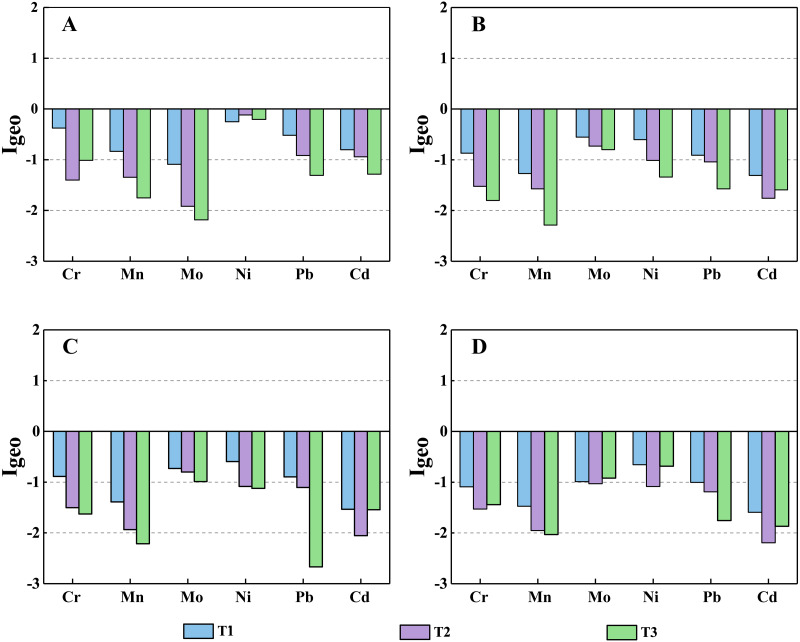
The I_geo_ values of HMs in three profiles at the depth of the 0–10 cm (A), 10–20 cm (B), 20–30 cm (C), and 30–50 cm (D).

The mean values of the geo-accumulation Index (I_geo_) of the six HMs at 0–10 cm, 10–20 cm, 20–30 cm, 30–50 cm depths under diverse land-uses are presented in Fig. 6. The I_geo_ values of HMs in soils were lower than 0 in most layers, indicating that the three soil profiles were possibly not polluted by anthropogenic source (Muller, 1969). However, comparing the distributions of HMs concentrations in the three profiles, the agricultural activities at the T2 profile and the goats’ grazing activities near the T3 profile show limited impact on the HMs in soil.

### Ecological risk assessment

**Figure 7:**
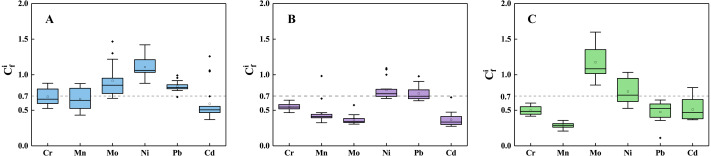
The contaminated factor of six HMs in the three profiles, including T1 profile (A), T2 profile (B), T3 profile (C).

The calculated contaminated factor (C_c_^i^) values of six HMs in the Yinjiang County are presented in Fig. 7. According to the classification of C_c_^i^ values by Qiu et al. (2016), the pollution degree of HMs in the research profile is only slight pollution at most, such as Ni in the T1 profile (the highest value: 1.42) and Mo in the T3 profile (the highest value: 1.60). Since the secondary forest has no disturbance from human activities, it can be speculated that the higher Ni concentrations in the T1 profile may correspond to natural factors such as the weathering of the parent rocks (Bonifacio, Falsone & Piazza, 2010). The pollution phenomenon that the high Ni concentrations in soils possibly resulted from the high background value of bedrock.

The enrichment of Mo in the surface layer of the T1 profile under secondary forest may be due to the plant uptake of Mo from subsurface soils (Brun et al., 2008) and return into surface soils through the plant litter fall (Marks et al., 2015). As the only profile where the C_c_^i^ values of almost all soils are greater than 1, the T3 profile may have received exogenous HMs input. The T3 profile has experienced intensive human activity (5-year grazing period) in recent years. Mo is often added into the feed, and most of Mo ingested by animals will be excreted with feces (Gooneratne et al., 1989; Ivan & Veira, 1985). Therefore, the animal feces may have more Mo which may migrate into deeper layers as a result of leaching processes. The result showed that only two C_c_^i^ values of Ni are higher than 1 in the T2 profile (the value in 110–120 cm: 1.07, the value in 120–130 cm: 1.09), which might be attributed to the leaching and accumulation (Domergue & Védy, 1992). It can be determined that there are no exogenous inputs of HMs in the T2 profile. The C_c_^i^ values of Mo and Cd at the soil layer of 0–15 cm depth in the T1 profile were higher than 1, which might be derived from atmospheric deposition (Zhang et al., 2016a).

**Figure 8:**
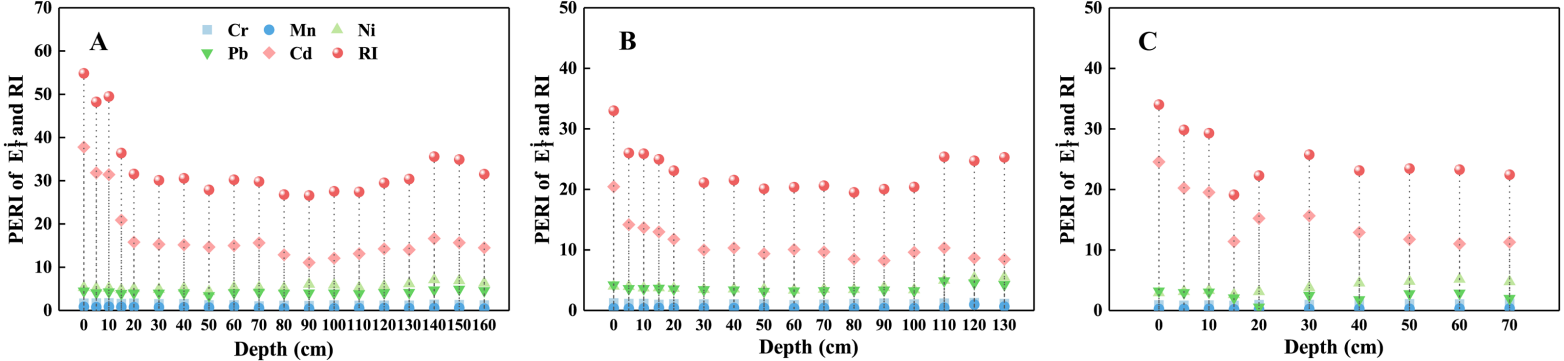
The potential ecological risks index of HMs in the three profiles, including T1 profile (A), T2 profile (B), T3 profile (C).

The comprehensive potential ecological risk index (RI) and the E_f_^i^ value of each HMs are presented in Fig. 8. Based on the mean values of E_f_^i^, the values of HMs follow the sequence: Cd > Ni > Pb > Cr > Mn in theT1 and T3 profiles, and Ni > Pb > Cd > Cr > Mn in the profile T2. According to the classification of RI from Hakanson (1980), the ecological risk in Yinjiang County soils is slight (RI < 60). Therefore, the overall quality of research profiles in the Yinjiang County is relatively safe. The management of land-use types in the study area is reasonable and the soil potential ecological risk is low.

## Conclusions

The HMs (Mn, Ni, Cr, Pb, Cd and Mo) concentrations were higher in the secondary forest land and those of abandoned cropland were higher than shrubland except Mo. The dominant influence factor of the distributions of most HMs may be the soil pH and SOC. The EF values of most samples were lower than 2 and the I_geo_ values were lower than 0 in the three profiles. This possibly indicates that the main source of HMs in study area is parent rocks instead of human activities. Results from PERI on the pollution degree and the potential ecological risk are also revealed that the quality of soils in the Yinjiang County is relatively safe. However, there is no great ecological risk under reasonable management. The multiple geographic analyses (I_geo_, C_c_^i^ and RI) of these HMs denoted the low ecological risk of the three profiles in the Yinjiang County. In addition, through the regulation of soil pH and the content of SOC, the content of HMs in soil can be controlled.

## Supplemental Information

10.7717/peerj.12716/supp-1 Supplemental Information 1 Raw data

## Additional Information and Declarations

### Data Availability

The following information was supplied regarding data availability: The raw measurements are available in the Supplementary Files.
